# Visual Acuity Testing: Feedback Affects Neither Outcome nor Reproducibility, but Leaves Participants Happier

**DOI:** 10.1371/journal.pone.0147803

**Published:** 2016-01-29

**Authors:** Michael Bach, Kerstin Schäfer

**Affiliations:** Section Visual Function / Electrophysiology Eye Center, Freiburg University Medical Center, Killianstr 5, 79106, Freiburg, Germany; State University of New York Downstate Medical Center, UNITED STATES

## Abstract

Assessment of visual acuity is a well standardized procedure at least for expert opinions and clinical trials. It is often recommended not giving patients feedback on the correctness of their responses. As this viewpoint has not been quantitatively examined so far, we quantitatively assessed possible effects of feedback on visual acuity testing. In 40 normal participants we presented Landolt Cs in 8 orientations using the automated Freiburg Acuity Test (FrACT, <michaelbach.de/fract. Over a run comprising 24 trials, the acuity threshold was measured with an adaptive staircase procedure. In an ABCDDCBA scheme, trial-by-trial feedback was provided in 2 x 4 conditions: (A) no feedback, (B) acoustic signals indicating correctness, (C)visual indication of correct orientation, and (D) a combination of (B) and (C). After each run the participants judged comfort. Main outcome measures were absolute visual acuity (logMAR), its test-retest agreement (limits of agreement) and participants’ comfort estimates on a 5-step symmetric Likert scale. Feedback influenced acuity outcome significantly (p = 0.02), but with a tiny effect size: 0.02 logMAR poorer acuity for (D) compared to (A), even weaker effects for (B) and (C). Test-retest agreement was high (limits of agreement: ± 1.0 lines) and did not depend on feedback (p>0.5). The comfort ranking clearly differed, by 2 steps on the Likert scale: the condition (A)–no feedback–was on average “slightly uncomfortable”, the other three conditions were “slightly comfortable” (p<0.0001). Feedback affected neither reproducibility nor the acuity outcome to any relevant extent. The participants, however, reported markedly greater comfort with any kind of feedback. We conclude that systematic feedback (as implemented in FrACT) offers nothing but advantages for routine use.

## Introduction

Assessing distant visual acuity is the procedure most frequently used to estimate visual performance. Because this is such an important and frequently performed test, it is relevant to make it as effective and comfortable as possible for participants, without confounding results.

One way to optimize the test would be to provide trial-by-trial feedback, however, some guidelines recommend not giving it [1], e.g. “The subject shall not be informed before the end of the test whether or not any mistakes were made” [2]. Acuity testing is a “type 1 experiment” [3], namely there is a “correct” and “incorrect” response, so feedback is possible. Yet we identified no reports assessing the effects of feedback on clinical acuity testing in a peer-reviewed publication. This is relevant, because for one, our own experience and anecdotal reports indicate that participants are more comfortable with feedback, and two, visual perceptive learning [4] also occurs in conjunction with visual acuity testing [5], is currently extensively assessed for modulating visual perceptual thresholds [6], and its effect size depends on feedback (around one line with feedback, half that value without). In other visual tasks, namely Vernier acuity testing, visual perceptual learning has been studied extensively: feedback accelerates learning [7,8] and incorrect feedback can prevent learning [9]. Although perceptual learning usually needs more trials than used in routine clinical testing, feedback could contribute to non-stationarity in acuity testing results.

Thus we designed this experiment to examine whether feedback influences the results, in a situation similar to routine clinical acuity assessment. The specific aims of this study were to quantify the influence of feedback on visual acuity’s absolute values, test-retest agreement, and the patients’ subjective comfort ranking.

## Materials and Methods

### Participants

**Fig 1:**
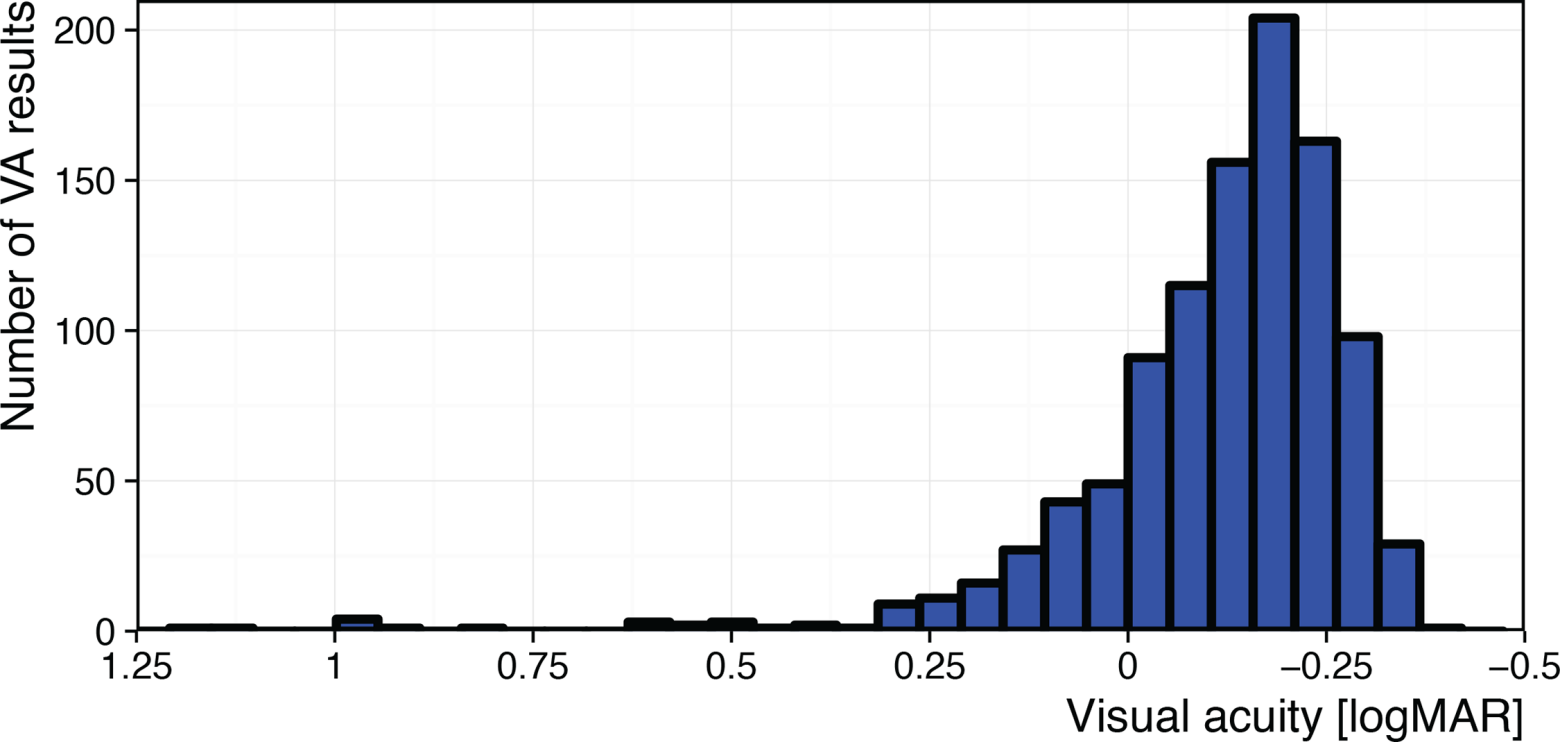
Distribution of visual acuities for all participants, eyes, test conditions and repeats; better acuity is to the right. Most acuities are in the normal range, with a few outliers. The poorest acuities (>0.75 logMAR) are all from a single eye in one participant.

We included both eyes from 40 research participants in a narrow age range (19–29 years, one 64 years). They wore their habitual correction; some individual acuities turned out to be quite low (Fig 1), but we did not view this as detrimental to the present question, rather we welcomed the greater range that entailed.

Prior to testing, all participants were informed about the study’s scientific purpose and the practices used to protect their data and right to privacy. Their informed consent was documented in written form. The survey design and the documentation form was approved by our local institutional review board (Ethik-Kommission der Albert-Ludwigs-Universität Freiburg, #64/12) and the study was performed in accordance with the ethical standards as laid down in the 1964 Helsinki declaration [10].

### Testing

The tests were carried out in an artificially lit room, with optotype and background luminance levels in accordance with the ISO standard on acuity testing [11]: Luminance of the optotype background was 120 cd/m^2^ and ambient illuminance was at 2 lux during all the test runs, concurring with the ISO [11] required range. The test distance was 4 m. Acuity was assessed via the Freiburg Acuity and Contrast Test (FrACT) [12,13], which was augmented for the study’s purposes with additional feedback options (version 3.8.1). FrACT is a computer program, freely available on-line [14] presenting optotypes in various sizes–we used Landolt Cs in the present study. Participants responded on a remote keypad by pressing one of eight buttons arranged spatially and marked in correspondence with the Landolt C’s 8 possible gap directions. The size of the optotypes followed an adaptive staircase procedure determined by a Best PEST [15]. The Best PEST “suggestions” are modified in two different ways: (1) the first three steps (assuming correct identification) follow the sequence VA_decimal_ = 0.1, 0.2, 0.4 (corresponding to logMAR 1.0, 0.7, 0.4) to comply with EN ISO 8596. (2) Steps 12, 18 and 24 were “easy trials”, that is an optotype 2.5× the current threshold estimate, to keep the participant motivated.

#### Test conditions

A–no feedback: on keypress, the presented optotype vanished, to be replaced after a 200 ms break with the next one, without any direct auditory or visual indication of whether the response was correct; here abbreviated as “none”.

B–acoustic feedback: In addition to (A), the response keypress elicited an audible tone whose quality indicated the correctness of response; abbreviated as “acoustic”.

C–visual feedback: In addition to (A), the response keypress, when incorrect, elicited a large red Landolt C, indicating the correct orientation and visible for 200 ms; abbreviated as “visual”

D–combination of (B) and (C), abbreviated as “both”.

Feedback was given on a trial-by-trial basis. Each of the four conditions was presented twice, following an ABCDDCBA scheme first for both eyes, then the right, then the left eye. Prior to these runs, the task was explained to the participants and warm-up trials were run as needed.

#### Rating of test comfort

Immediately after each run, the participant specified the level of comfort during the run by ticking a column on a chart with a 5-step symmetric Likert-like scale [16]. The five rating levels were as follows:

–2: Very uncomfortable (in German “sehr unangenehm”)
–1: Uncomfortable (“unangenehm”)
0: Neutral
1: Comfortable (“angenehm”)
2: Very comfortable (“sehr angenehm”)

### Data

Full anonymized data is deposited at http://dx.doi.org/10.6084/m9.figshare.2062785 including all analysis routines. The data set is in the file “allDataMinimal.xlsx”, the file “main.R” reproduces all figures using the open source R statistical programming environment [17] [the latter was used for all analysis reported here]. Test-retest agreement was quantified with the limits of agreement (LOA) [18]:

$$LOA=1.96\cdot mean\;\left(\frac{{SD}_{i}}{m_{i}}\right)$$ (1)

Since results from the two eyes will correlate closely, the “eyes or patients” problem arises. Test-retest agreement, however, is unaffected by this; for the ANOVA on acuity outcome, we accounted for this with a mixed design with eyes as a “within” factor. For the participants’ ratings, for quantitative analysis with R’s “likert” package the results were averaged per participant over eye and run, and a Kruskal-Wallis test instead of ANOVA employed to take account of the non-normal distribution of a rating scale.

## Results

Fig 1 illustrates the distribution of acuity outcomes across all eyes and conditions. Most acuities are ≤ 0.0 logMAR as expected for normal participants. The outliers are probably due to inadequate habitual correction; the poorest acuities (>0.75 logMAR) were all from a single eye in one participant.

### Acuity results across conditions

**Fig 2:**
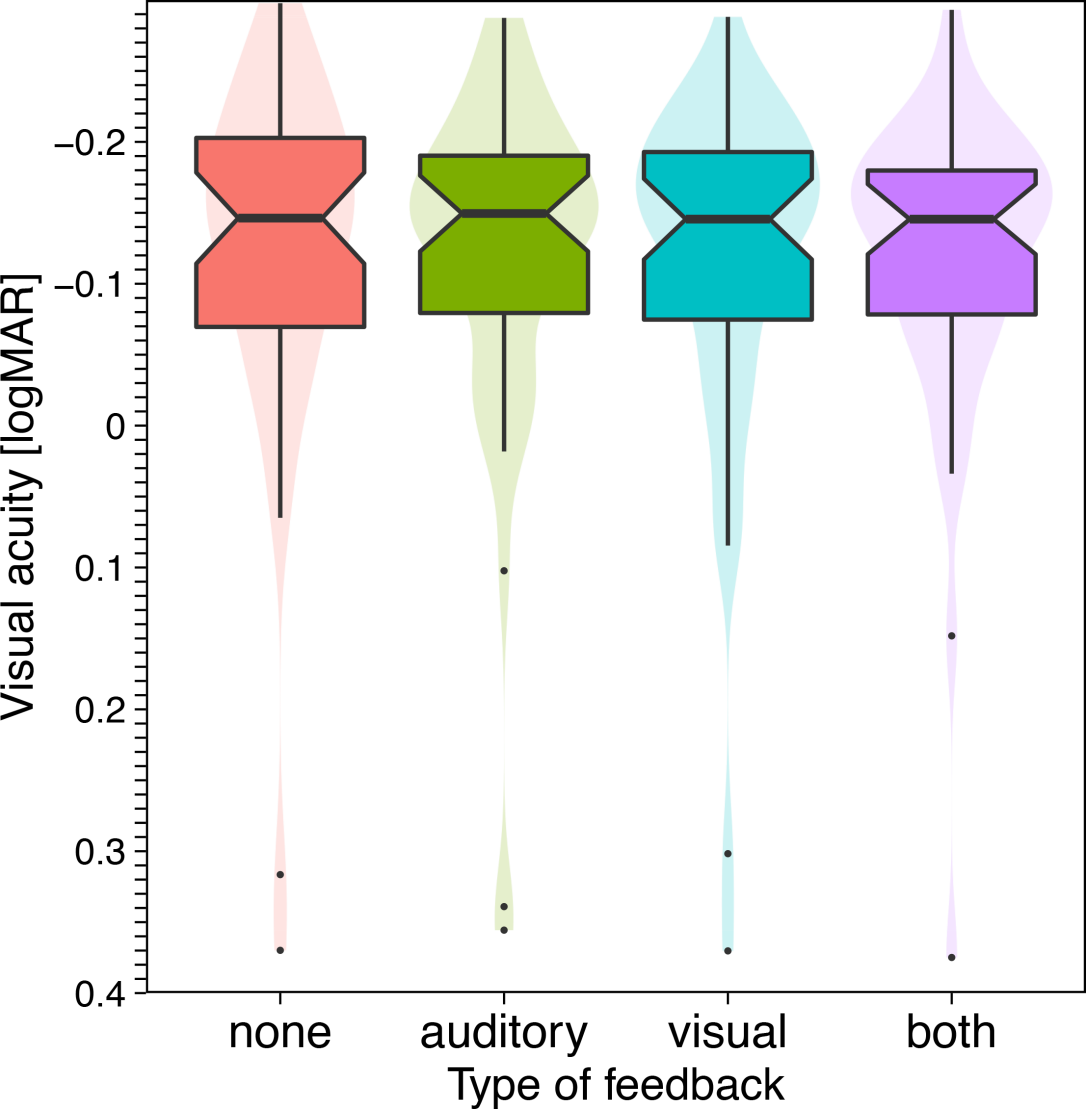
Acuity results (in logMAR, negative values at the top) across the four different feedback conditions. Most acuities are between –0.1 and –0.2 logMAR, and thus normal, but there are low acuity outliers. The feedback condition has no noticeable effect on visual acuity. [Box plot details: thick horizontal bar: median. Notch: 95% confidence interval of the median. Box: interquartile range (25%–75%). Whiskers: range. Dots: outliers (data >1.5 times the interquartile range off the box). The ‘‘violin plots” in the background visualize a smoothened density estimate.]

**Fig 3:**
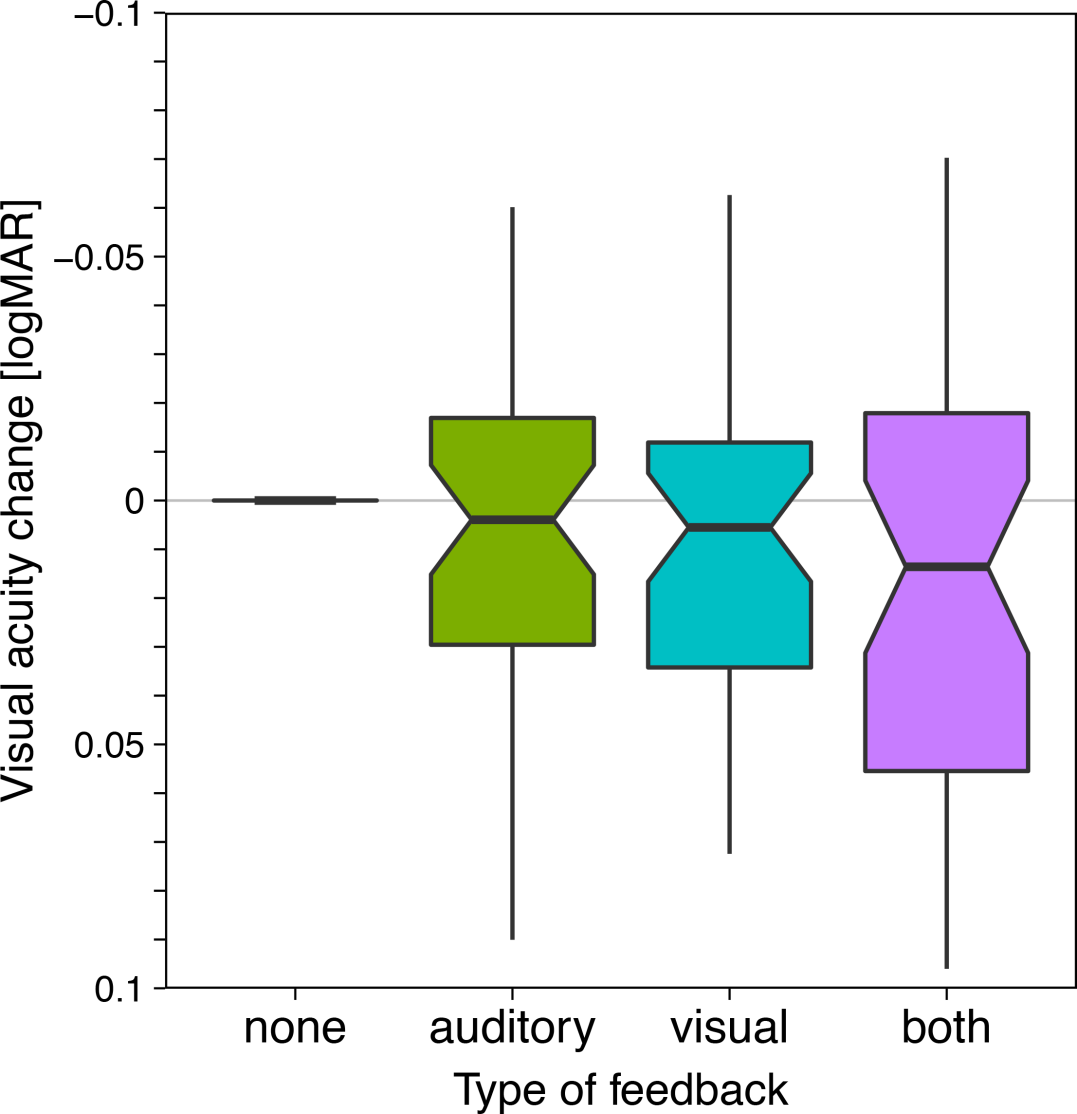
Acuity results across the four different feedback conditions. Details as in Fig 2, but here the values are normalized with respect to the “none” condition, allowing scale expansion. Note that for the “both” condition, slightly lower acuity resulted; the small difference of 0.02 logMAR is, however, clinically irrelevant.

Fig 2 displays acuity results for the four feedback conditions. The few acuity outliers are obvious, and there is no visible influence from feedback on the results. For closer inspection, Fig 3 displays these results in a normalized fashion: for every condition, the logMAR acuity for the the “none” condition was subtracted. Now a slightly lower acuity for the “both” condition becomes apparent. The maximum differences between conditions are below 0.02 logMAR. These observations are borne out statistically. A repeated measures ANOVA (acuity ~ condition × take) revealed a significant influence of condition (p = 0.017) and take (p = 0.0043) without interaction. The factor “take” (first test vs. second test) will be discussed in the section on test-retest agreement.

### Test-retest agreement across conditions

**Fig 4:**
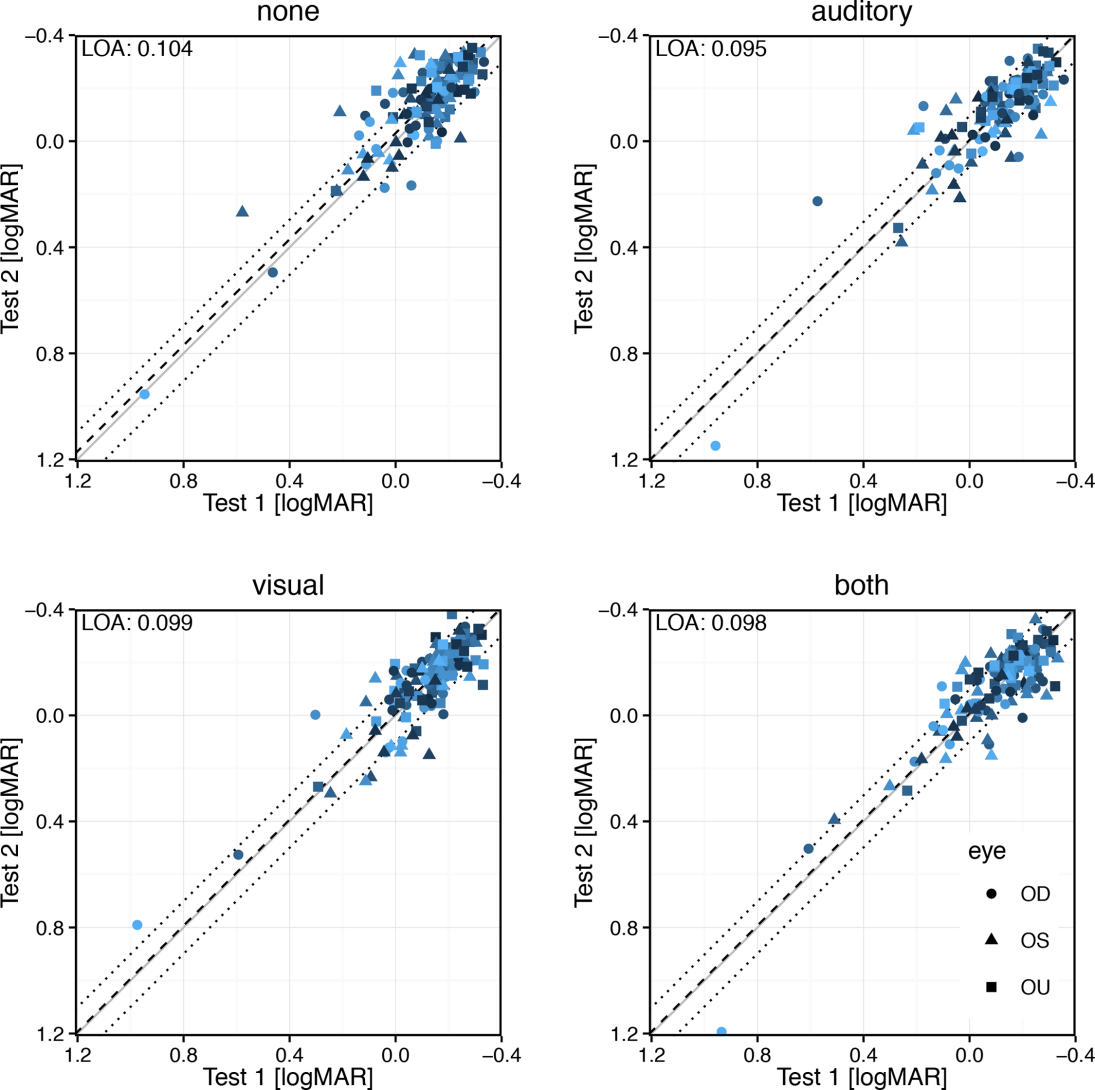
Test-retest agreement across feedback conditions. The identity line is indicated by a solid gray line. Symbol color varies by participant to better unconfound overplotting. The dashed line represents the mean difference, the dotted lines the limits of agreement (LOA, Eq (1)) which are also enumerated at top left. The LOAs are quite low (≈0.1 logMAR) and closely similar across conditions. Thus the type of feedback does not influence test-retest agreement. The mean differences are always slightly above the identity line, indicating a slight learning effect (see Discussion).

In Fig 4 the test-retest agreement can be assessed. There is a sizable difference apparent between conditions. This is borne out numerically: the limits of agreement lie between 0.095 and 0.100 logMAR. They are thus quite low (about ±1 line) and very similar across conditions. The mean differences (dashed lines in Fig 4) are slightly above zero, and significantly so, as indicated by the significant (p = 0.0043) influence of the factor “take” (test 1 vs. test 2) in the ANOVA. Thus on average acuity results improved slightly over time. The grand mean difference between the first and second take was 0.0123 logMAR.

### Participants’ ratings across conditions

**Fig 5:**
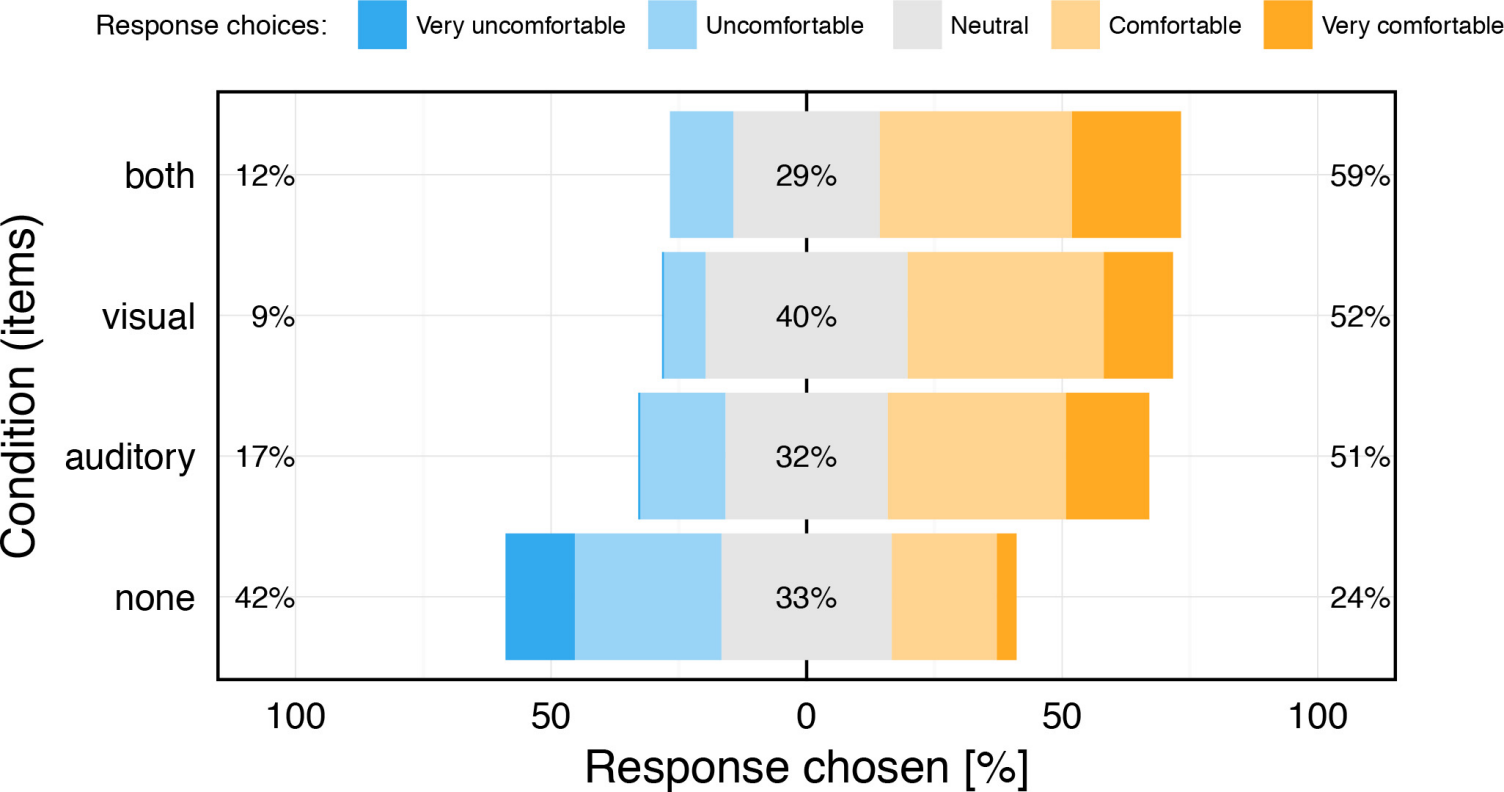
Likert plot of the participants’ rating of the test, compared across feedback conditions. The “none” condition (bottom) leaves the participants in a somewhat negative mood, while any type of feedback is evaluated positively (p<10^−7^).

Our participants rated the testing experience after each run in 5 categories, from –2 (negative) over 0 (neutral) to +2 (positive). In Fig 5 it can be seen that, in contrast to acuity outcome and test-retest agreement, feedback does have an effect on the test participants’ evaluation: any type of feedback is evaluated positively compared to the condition “none”. The Kruskal-Wallis rank sum test revealed a highly significant effect of feedback on rating (p<10^−7^). Post-hoc tests found highly significant differences between “none” and each of the three feedback other conditions (always p<10^−5^), remaining significant after Bonferroni correction. There were no significant differences between the other conditions (smallest p = 0.12 after Bonferroni-Holm correction).

## Discussion

This study was designed to assess the effect of trial-by-trial feedback on visual acuity outcome, test-retest agreement, and participants’ comfort. All in all, there was no clinically relevant effect on either visual acuity outcome or variability, but any type of feedback was clearly preferred over no feedback.

### Absolute acuity values across conditions

In Fig 2 there appears to be no difference in acuity across conditions. After normalization, however, Fig 3 shows that feedback results in slightly lower acuity, especially for the “both” condition; significant with p = 0.017. The actual difference, however, is ≤0.02 logMAR, corresponding to ≤0.2 lines (a one-line difference corresponds to 0.1 logMAR). This tiny effect is not clinically relevant.

### Test-retest agreement across conditions

The limits of agreement were identical within a 5% range across all conditions, around 0.1 logMAR. This is surprisingly low and identical to the value reported by Arditi & Cagenello [19] for trained research participants.

### Visual perceptual learning

Visual perceptual learning clearly exists [4,20]. In this context, “fast learning” [21,22] corresponds to several blocks of 40 or 50 trials each. Heinrich et al. [5] detected perceptual learning with Landolt-C visual acuity–with feedback: 0.04 logMAR after the first block of 50 trials, no feedback: ≈0.02 after 50 trials (from their Fig 4, the effect size rose markedly after further blocks). In the present study, 2 × 24 × 4 trials were presented, so it is likely that some perceptual learning also occurred, and it would be faster or stronger with feedback. Indeed, across all pairs of identical conditions the respective second run resulted in slightly better acuity by about 0.01 logMAR. We conclude that visual perceptual learning did indeed occur during this experiment, albeit to a clinically irrelevant degree (about 1/10^th^ of a line). For routine clinical testing, where an 18-trial binocular run would be run for training and then just one more 18-trial run per eye, effects would be even lower. All in all, perceptual learning’s acceleration through feedback would not affect visual acuity outcome by any relevant amount.

### Participant comfort rating across conditions

We noted a clear feedback effect on the comfort ratings, indicating significantly and markedly greater comfort with any feedback versus none. At the test’s beginning, some participants reported having trouble with how the the levels were classified, resolved after a few warm-up trials. Most participants informally reported that feedback motivated them to achieve the best possible result, although it did not actually affect acuity outcome–as is typical for psychophysical threshold situations, where the subjective impression correlates little with the behavioral outcome. From our own experience with FrACT, we fully agree that the testing situation is at least less uncomfortable with feedback–threshold regions never feel really comfortable.

### Limitations and generalizability

There are various limitations on our findings’ generalizability.

- We intentionally concentrated on a narrow age range (all but one withing 19–29 years) to remove age as a confounding factor, which begs the question whether we would have obtained these findings in elderly subjects. While we believe this is likely, it requires examination in future studies.
- We used the ISO-recommended Landolt C as optotype. Very frequently, other optotypes, namely letters, are employed [23]. Again, while we deem it likely, generalizability of the present findings to letters needs to be ascertained.
- Routine clinical assessment of visual acuity is often subject to time constraints and done less formally than prescribed while still adhering to the pertinent ISO norm. Furthermore, optotypes are announced and patients are motivated verbally. If feedback were given as “look a little more closely” (even allowing the participant to change her response based on a consequentially narrower range of choices), the outcome’s validity could be adversely affected. Thus the present findings may be somewhat specific to the automated acuity assessment as performed by FrACT.

## Conclusions

Trial-by-trial feedback was generally welcomed by our participants, and it should enhance patients’ comfort when employed in a clinical setting. Using the automatic feedback options in the Freiburg Test “FrACT” did not affect visual acuity outcome to any relevant degree. Thus neutral feedback that does not manipulate patients’ strategies appears to benefit the routine clinical assessment of visual function. Future studies should address generalizability over the entire age range, to other optotypes (e.g., Sloan letters), and measurement protocols other than “FrACT”.
